# Supplementary material for: Thiosemicarbazones Can Act Synergistically with Anthracyclines to Downregulate CHEK1 Expression and Induce DNA Damage in Cell Lines Derived from Pediatric Solid Tumors
Source: Int J Mol Sci. 2022 Aug 1;23(15):8549. doi: 10.3390/ijms23158549 (PMC9369312; doi:10.3390/ijms23158549)
Supplement: Supplementary file 1 [file ijms-23-08549-s001.zip › ijms-1822621-supplementary.pdf]

SUPPLEMENTARY FIGURE S1

Experiment 1:

Pgp

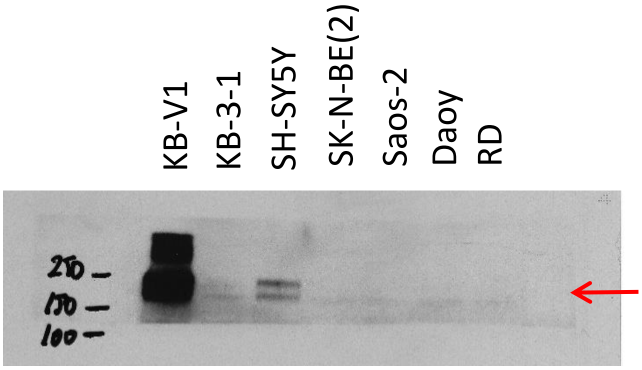

GAPDH

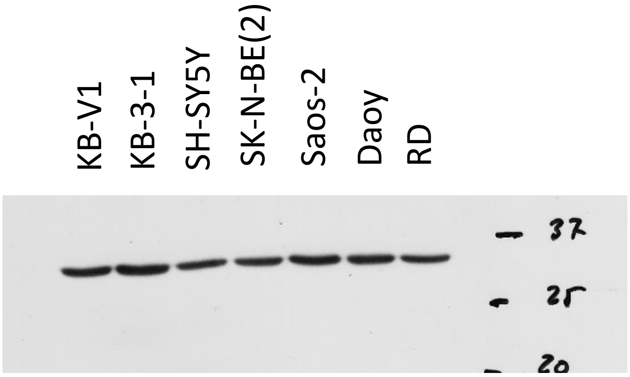

Experiment 2:

Pgp

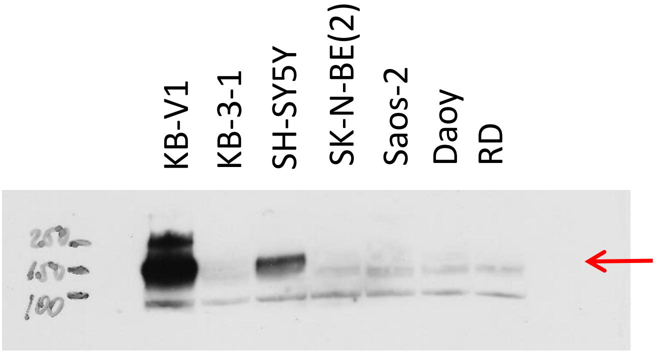

GAPDH

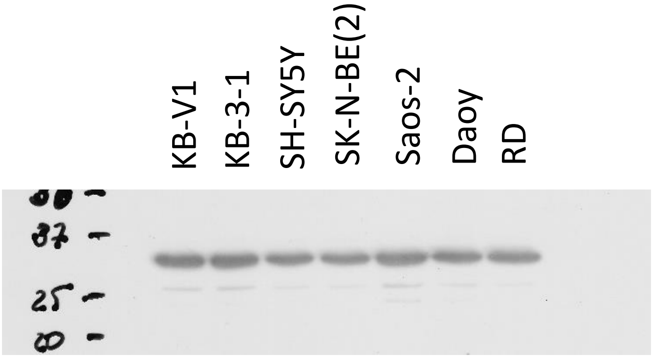

Experiment 3:

Pgp

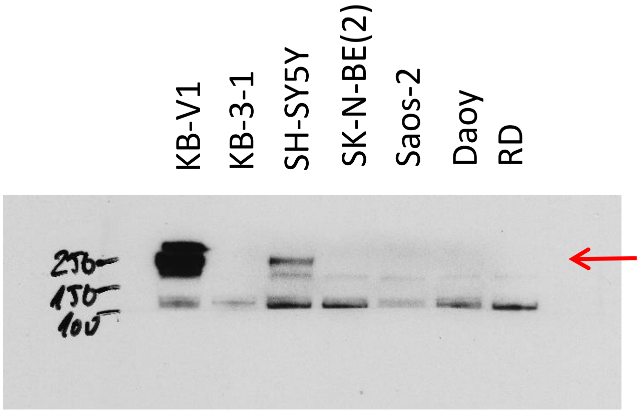

GAPDH

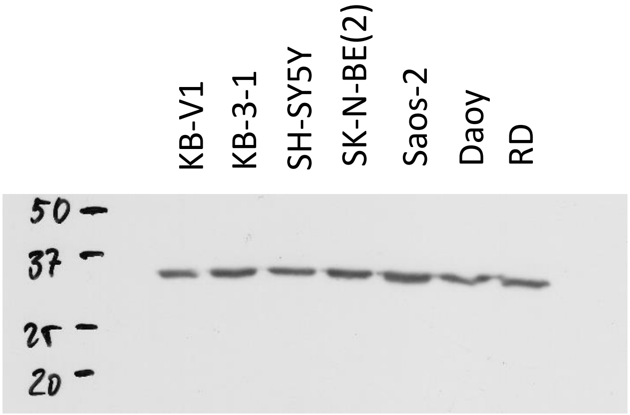

Figure S1: Immunoblotting to determine endogenous Pgp levels in untreated cell lines. GAPDH served as the loading control. The experiments were performer in biological triplicates.

SUPPLEMENTARY FIGURE S2

(A) KB-V1 cell line (positive control)

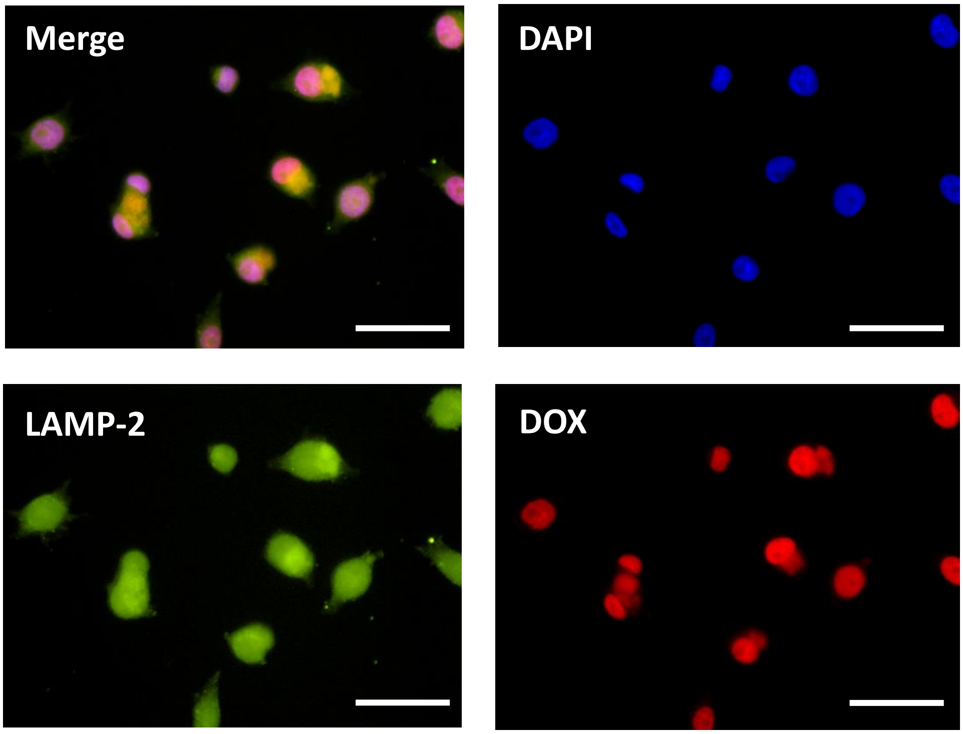

(B) KB-3-1 cell line (negative control)

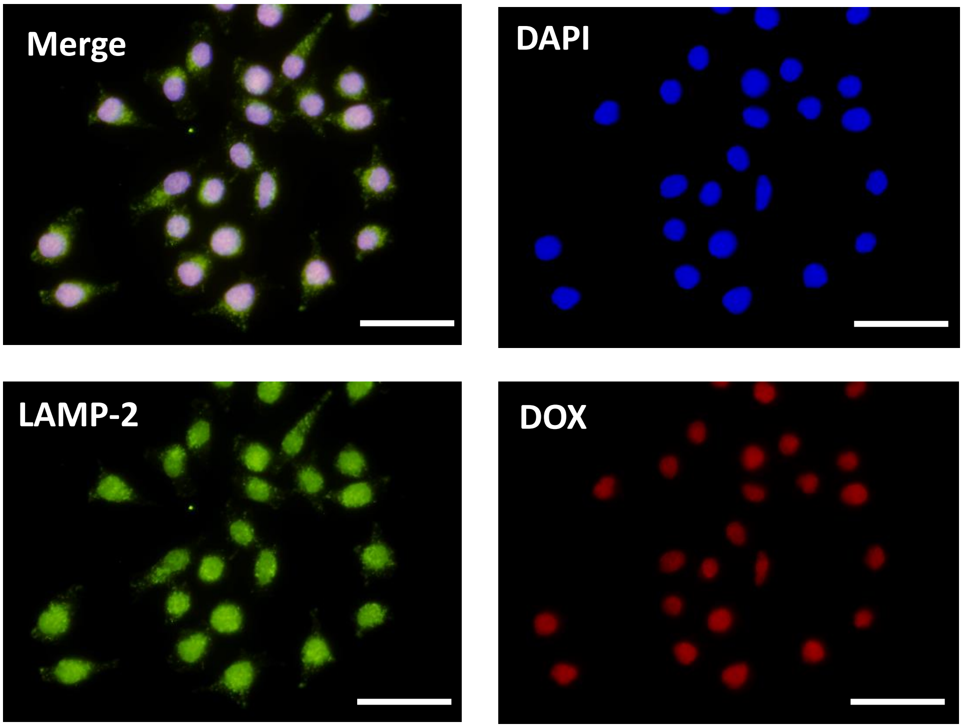

Figure S2: Intracellular localization of doxorubicin. The localization of doxorubicin (DOX) within the cells after 2 h of treatment with 10 μM DOX (red) was observed using immunofluorescence microscopy. The target organelles, lysosomes and nuclei, were also stained. The lysosomes were visualized by indirect immunofluorescence using LAMP-2 primary antibody and Alexa 488-conjugated secondary antibody (green). The nuclei were labeled with DAPI (blue). **(A)** KB-V1 cell line served as a positive control for the lysosomal sequestration of DOX; **(B)** KB-3-1 cell line served as a negative control. Scale bars, 50 μm.

SUPPLEMENTARY FIGURE S2

(C) SH-SY5Y cell line

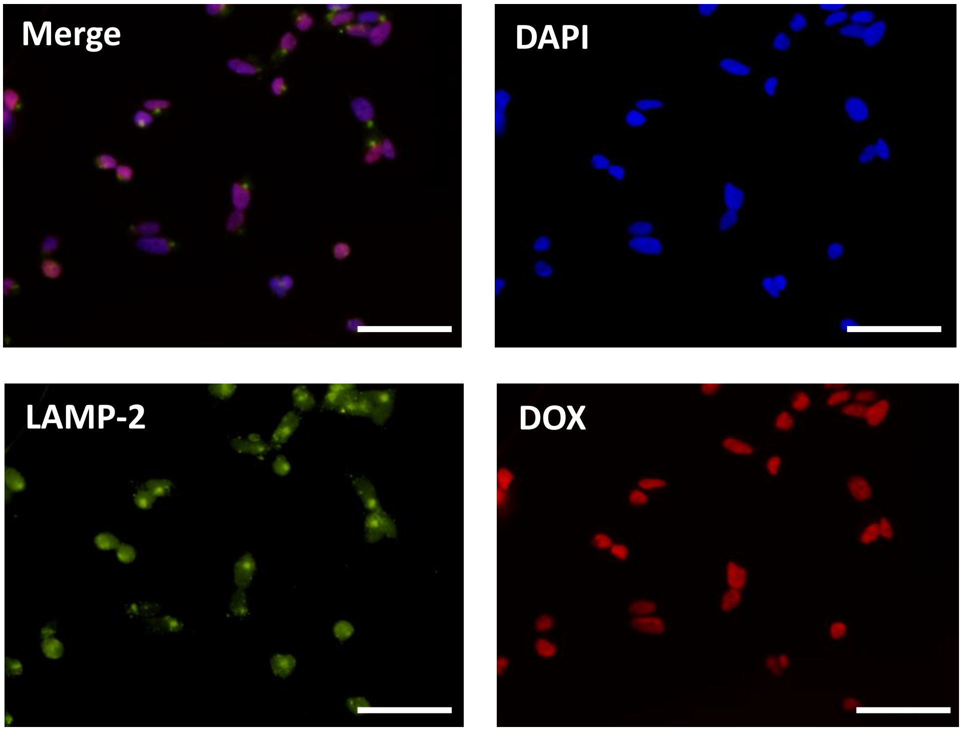

(D) SK-N-BE(2) cell line

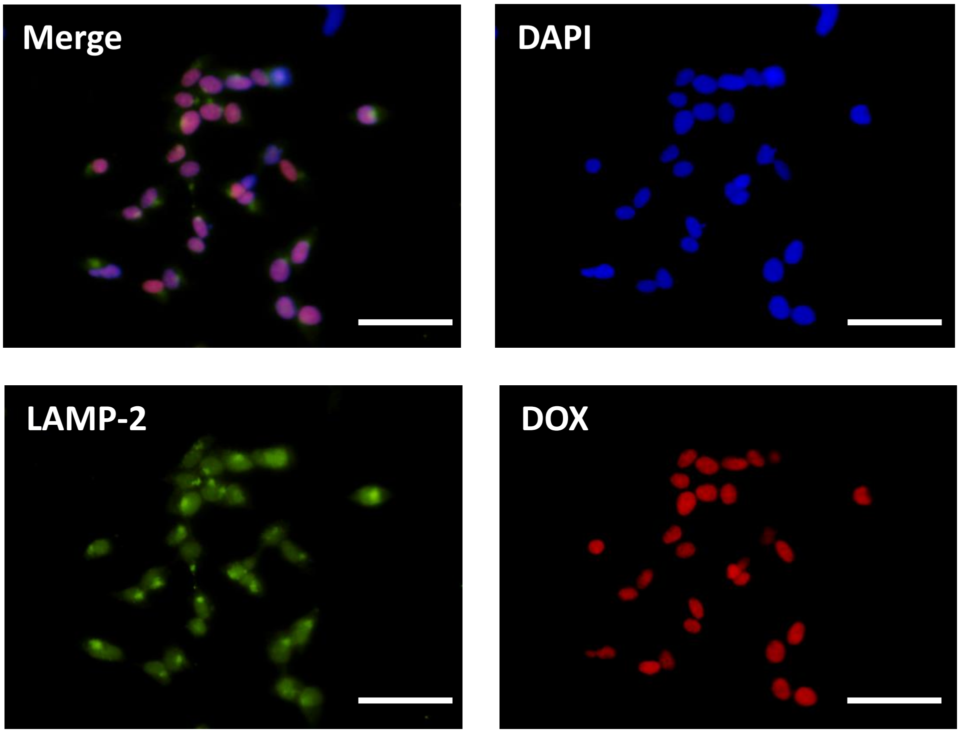

Figure S2: Intracellular localization of doxorubicin. The localization of doxorubicin (DOX) within the cells after 2 h of treatment with 10 µM DOX (red) was observed using immunofluorescence microscopy. The target organelles, lysosomes and nuclei, were also stained. The lysosomes were visualized by indirect immunofluorescence using LAMP-2 primary antibody and Alexa 488-conjugated secondary antibody (green). The nuclei were labeled with DAPI (blue). Scale bars, 50 µm.

SUPPLEMENTARY FIGURE S2

(E) Saos-2 cell line

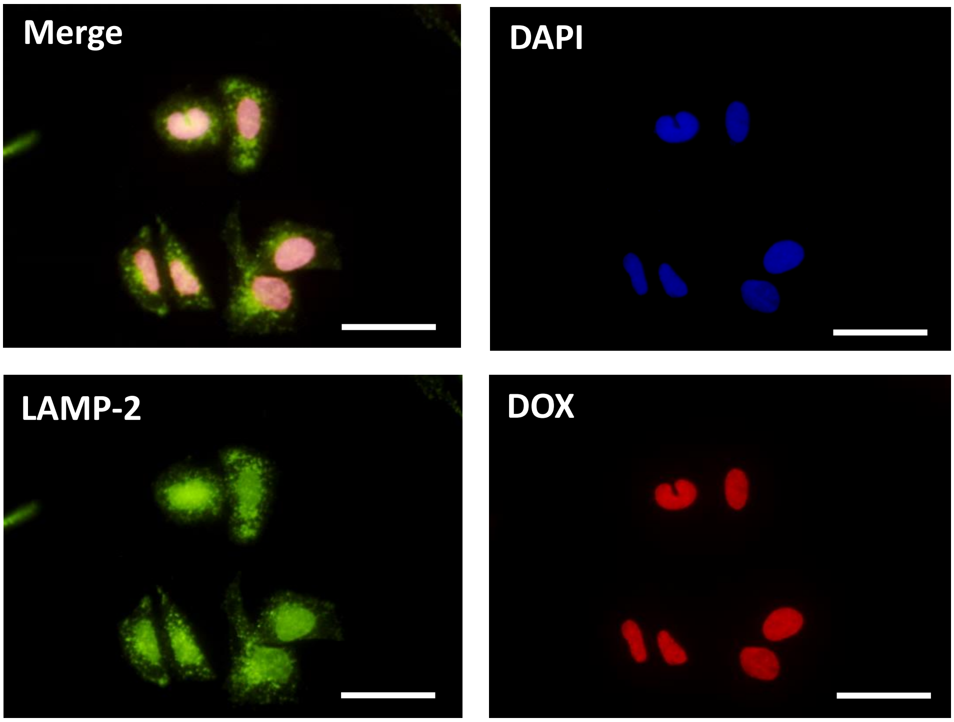

(F) Daoy cell line

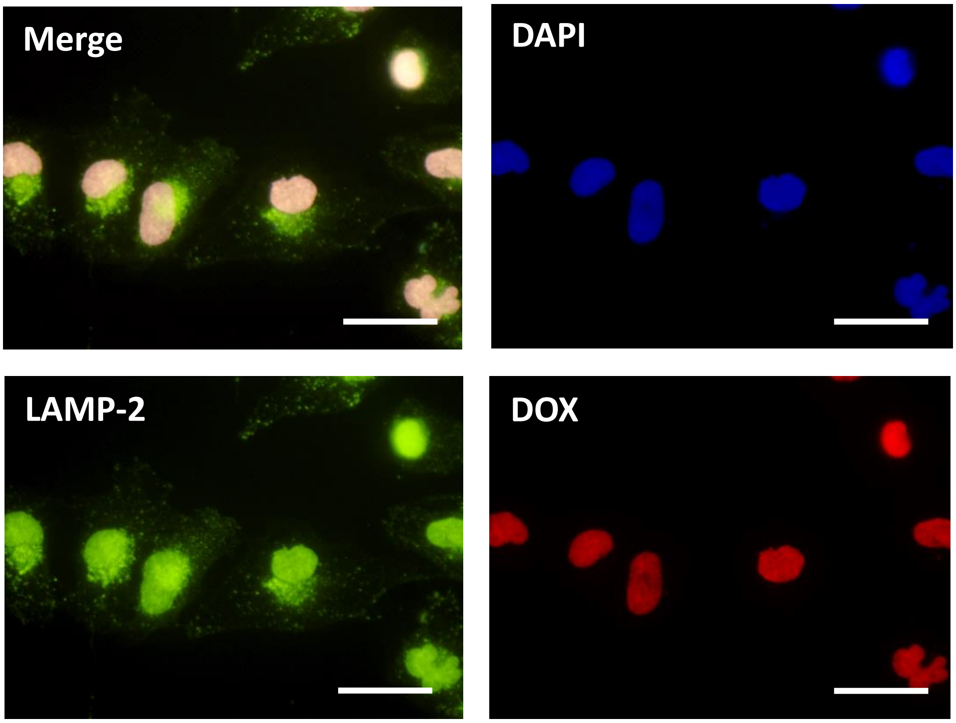

Figure S2: Intracellular localization of doxorubicin. The localization of doxorubicin (DOX) within the cells after 2 h of treatment with 10 μM DOX (red) was observed using immunofluorescence microscopy. The target organelles, lysosomes and nuclei, were also stained. The lysosomes were visualized by indirect immunofluorescence using LAMP-2 primary antibody and Alexa 488-conjugated secondary antibody (green). The nuclei were labeled with DAPI (blue). Scale bars, 50 μm.

SUPPLEMENTARY FIGURE S2

(G) RD cell line

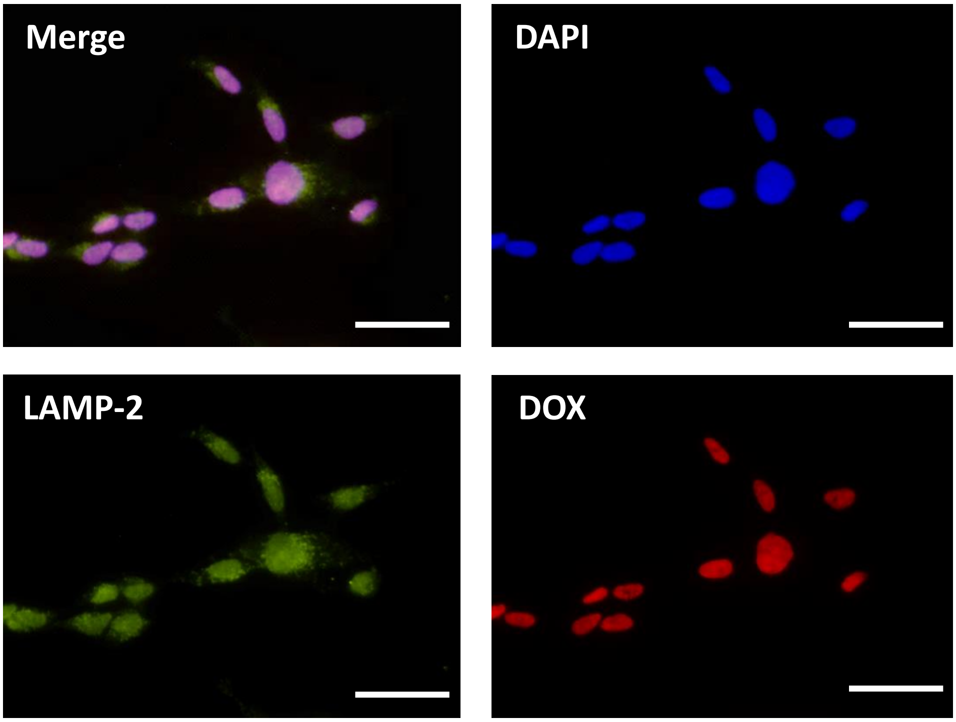

Figure S2: Intracellular localization of doxorubicin. The localization of doxorubicin (DOX) within the cells after 2 h of treatment with 10 µM DOX (red) was observed using immunofluorescence microscopy. The target organelles, lysosomes and nuclei, were also stained. The lysosomes were visualized by indirect immunofluorescence using LAMP-2 primary antibody and Alexa 488-conjugated secondary antibody (green). The nuclei were labeled with DAPI (blue). Scale bars, 50 µm.

SUPPLEMENTARY FIGURE S3

(A) SH-SY5Y cell line

Experiment 1

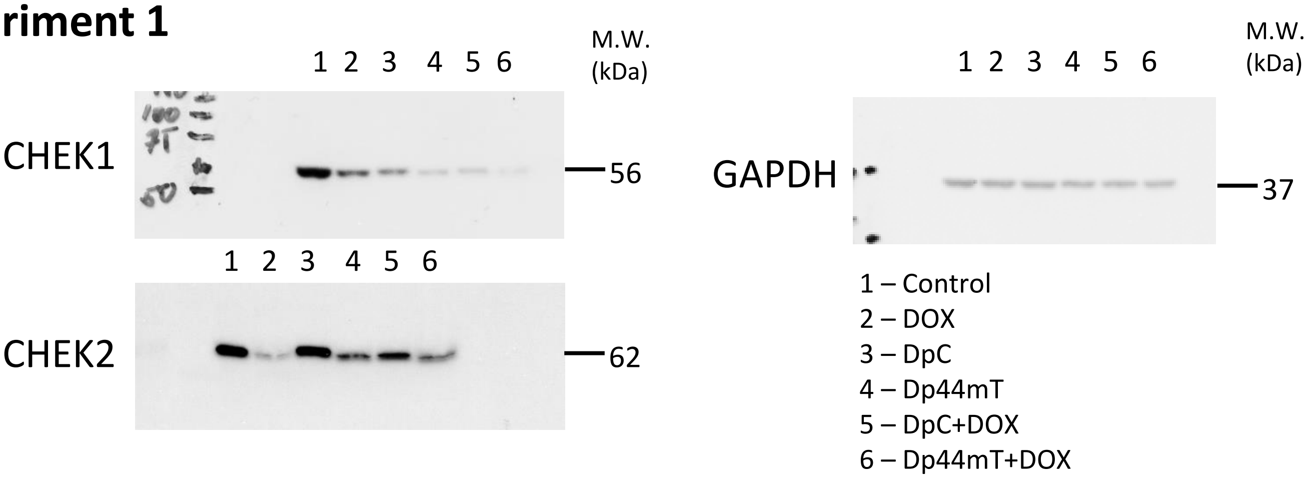

Experiment 2

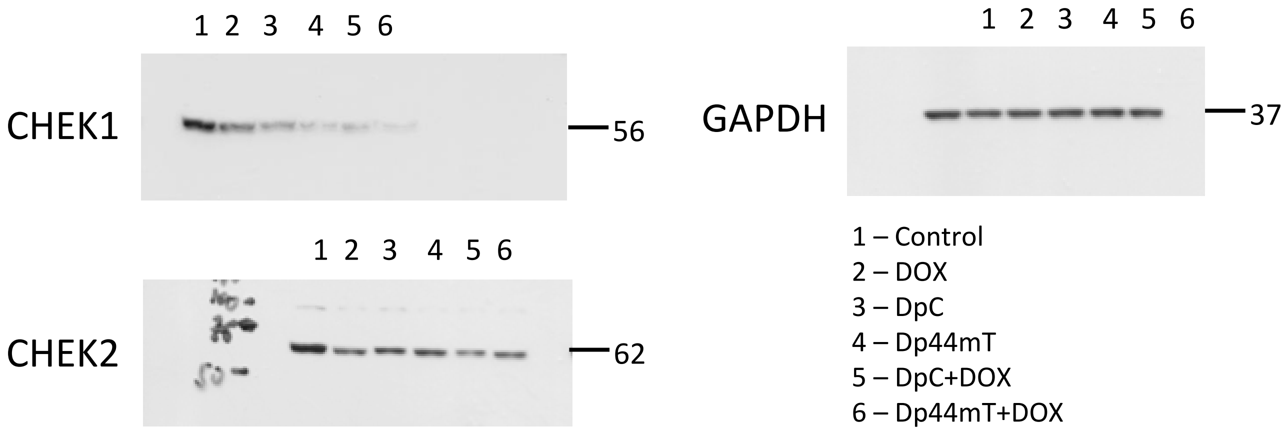

Experiment 3

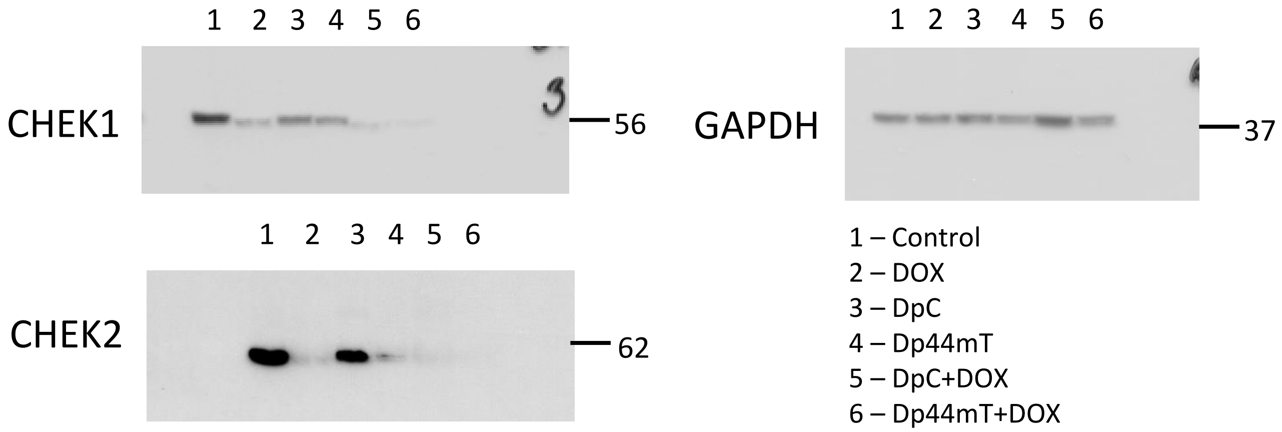

Figure S3: Immunoblotting to determine CHEK1 and CHEK2 levels in cell lines after the experimental treatment. CHEK1 and CHEK2 levels were examined after 3 days of incubation with IC_{50} doses of DOX, DpC, Dp44mT or their combinations. GAPDH served as the loading control. The experiments were performer in biological triplicates.

SUPPLEMENTARY FIGURE S3

(B) SK-N-BE(2) cell line

Experiment 1

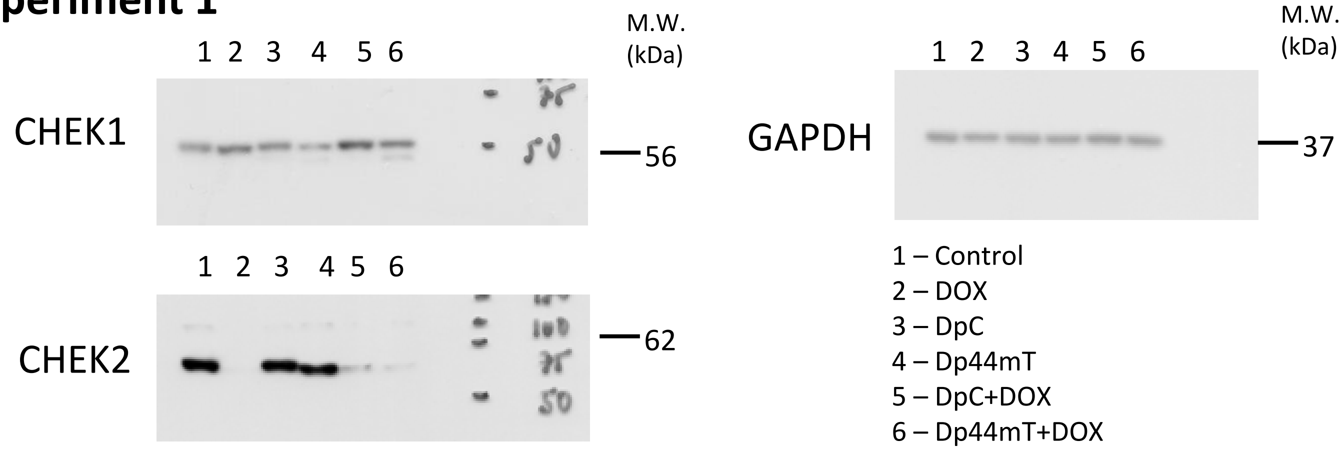

Experiment 2

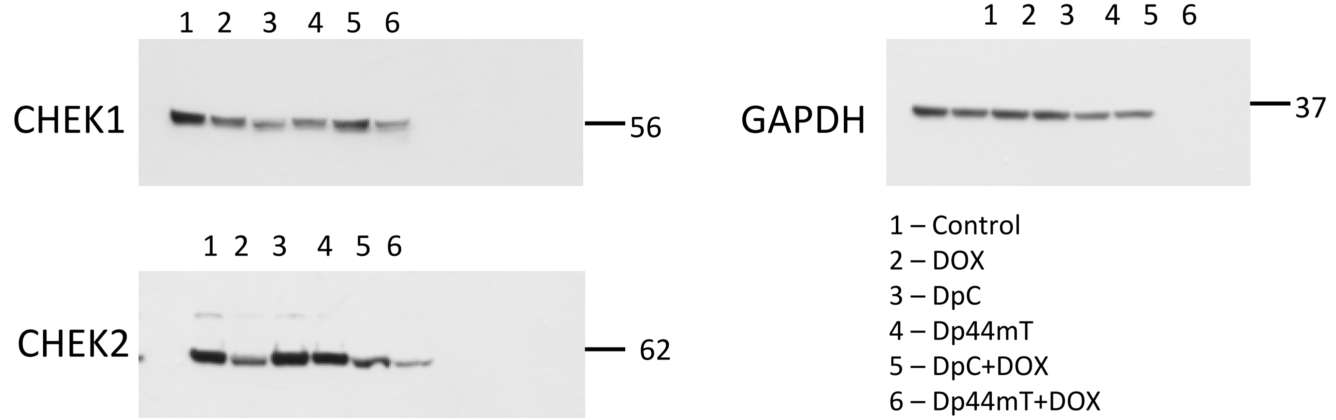

Experiment 3

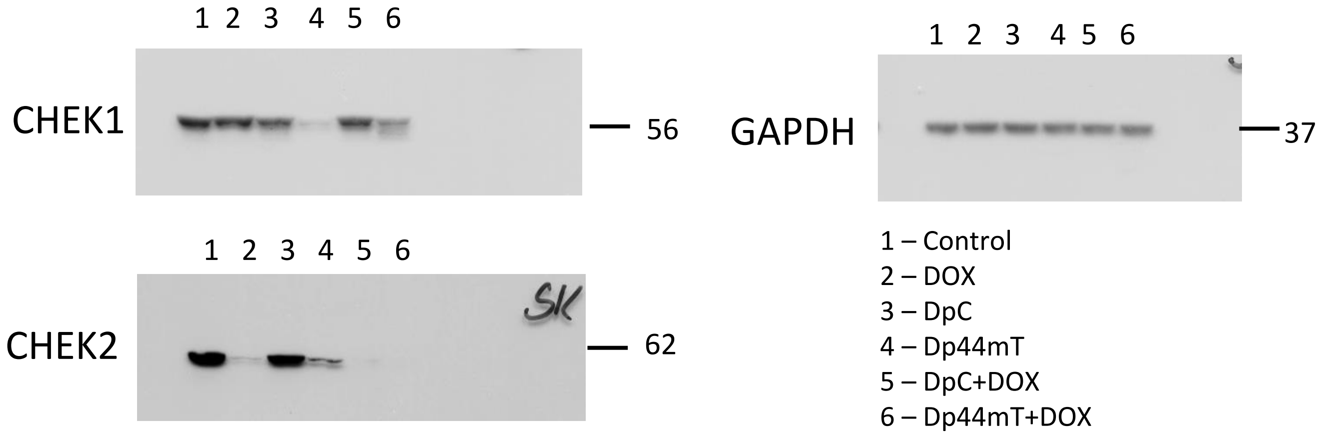

Figure S3: Immunoblotting to determine CHEK1 and CHEK2 levels in cell lines after the experimental treatment. CHEK1 and CHEK2 levels were examined after 3 days of incubation with IC_{50} doses of DOX, DpC, Dp44mT or their combinations. GAPDH served as the loading control. The experiments were performer in biological triplicates.

SUPPLEMENTARY FIGURE S3

(C) Saos-2 cell line

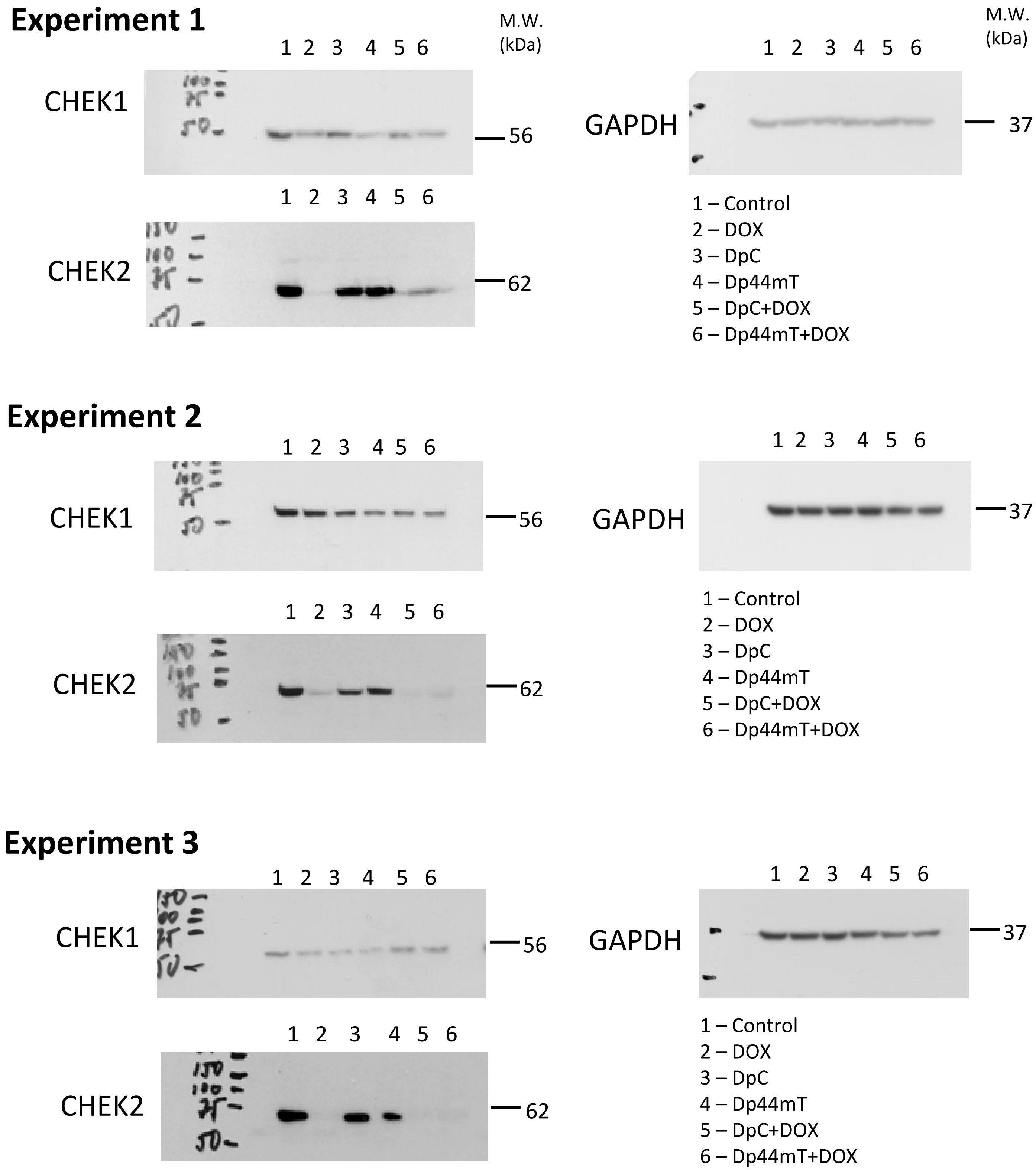

Figure S3: Immunoblotting to determine CHEK1 and CHEK2 levels in cell lines after the experimental treatment. CHEK1 and CHEK2 levels were examined after 3 days of incubation with IC_{50} doses of DOX, DpC, Dp44mT or their combinations. GAPDH served as the loading control. The experiments were performer in biological triplicates.

SUPPLEMENTARY FIGURE S3

(D) Daoy cell line

Experiment 1

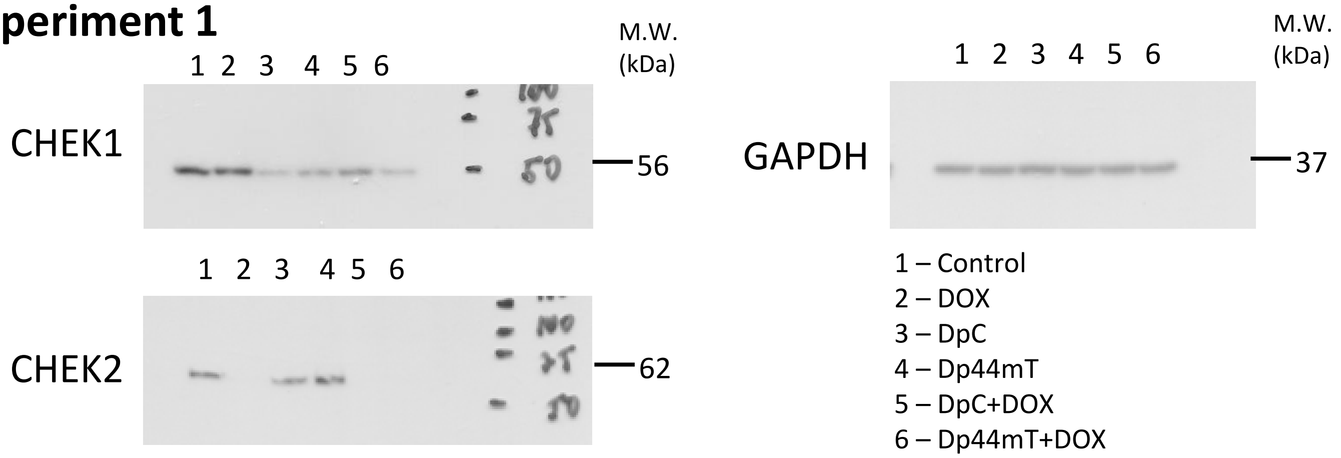

Experiment 2

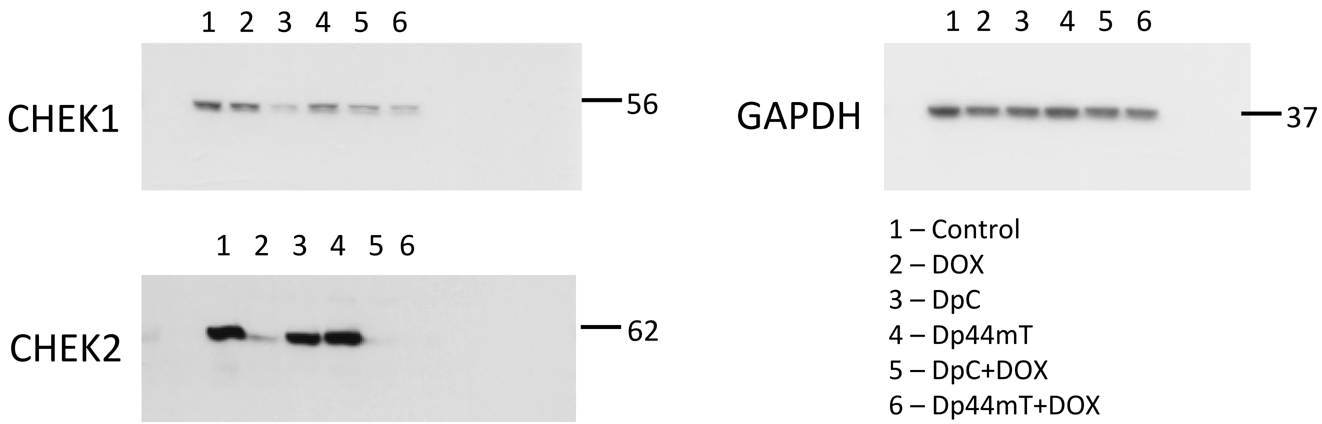

Experiment 3

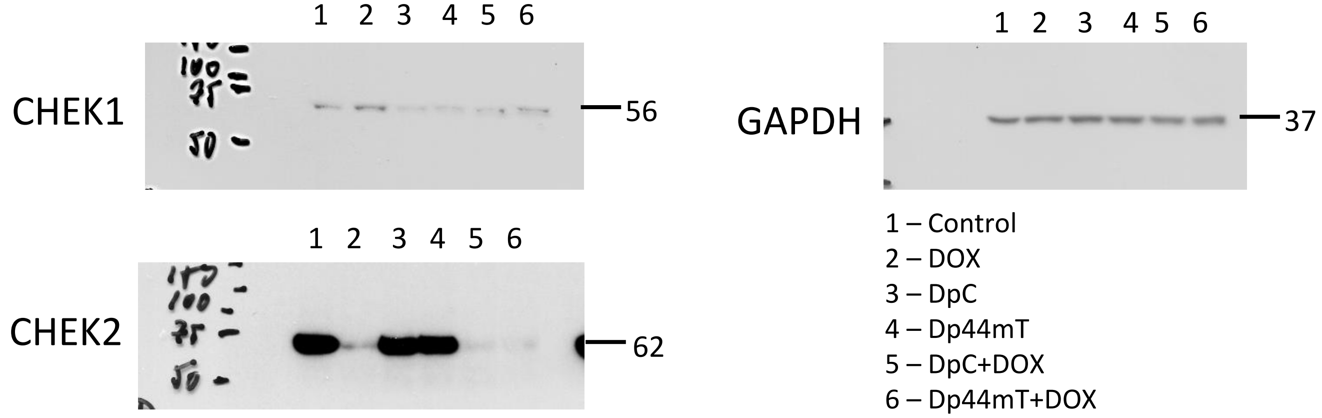

Figure S3: Immunoblotting to determine CHEK1 and CHEK2 levels in cell lines after the experimental treatment. CHEK1 and CHEK2 levels were examined after 3 days of incubation with IC_{50} doses of DOX, DpC, Dp44mT or their combinations. GAPDH served as the loading control. The experiments were performer in biological triplicates.

SUPPLEMENTARY FIGURE S3

(E) RD cell line

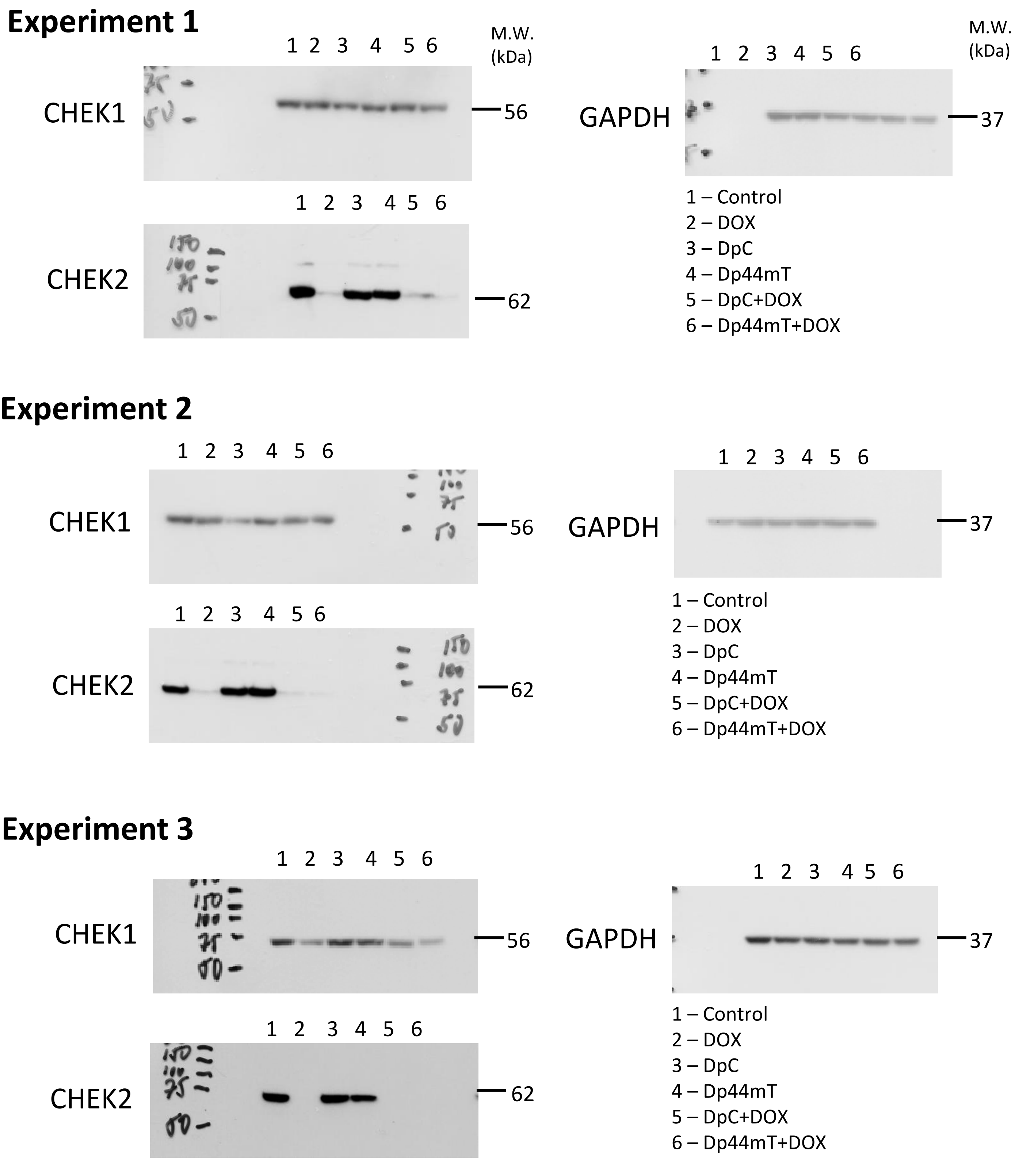

Figure S3: Immunoblotting to determine CHEK1 and CHEK2 levels in cell lines after the experimental treatment. CHEK1 and CHEK2 levels were examined after 3 days of incubation with IC_{50} doses of DOX, DpC, Dp44mT or their combinations. GAPDH served as the loading control. The experiments were performer in biological triplicates.

SUPPLEMENTARY FIGURE S4

(A) SH-SY5Y cell line

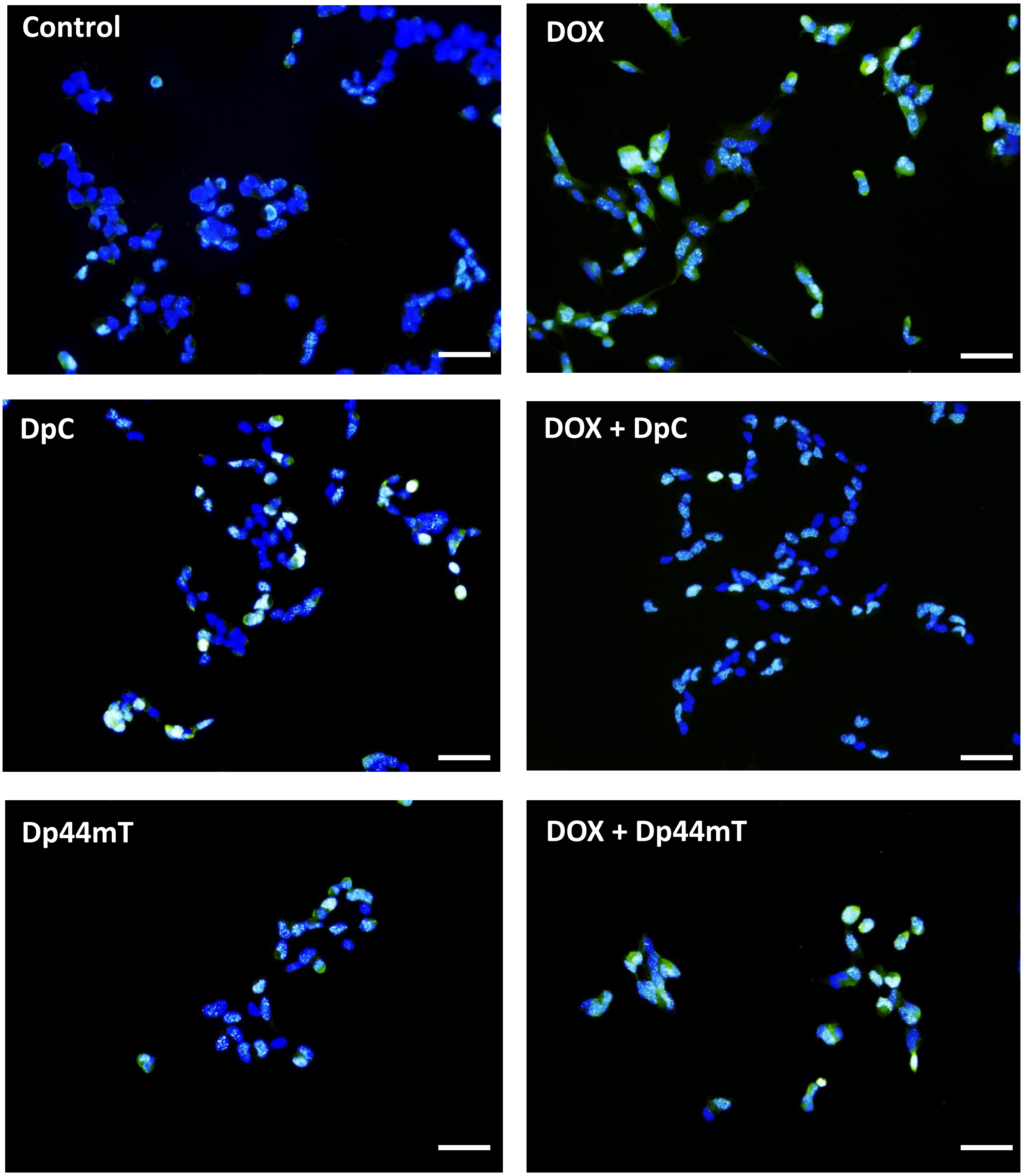

Figure S4: Detection of phosphorylated γ-H2AX as double-strand break marker in cell lines after the experimental treatment. Immunofluorescence was performer after 48 h treatment with IC_{50} doses of DpC, Dp44mT, DOX or their combinations. Phosphorylated γ-H2AX was visualized using the γ-H2AX primary antibody and Alexa 488-conjugated secondary antibody (green). The nuclei were labeled by DAPI (blue). Scale bars, 50 µm.

SUPPLEMENTARY FIGURE S4

(B) SK-N-BE(2) cell line

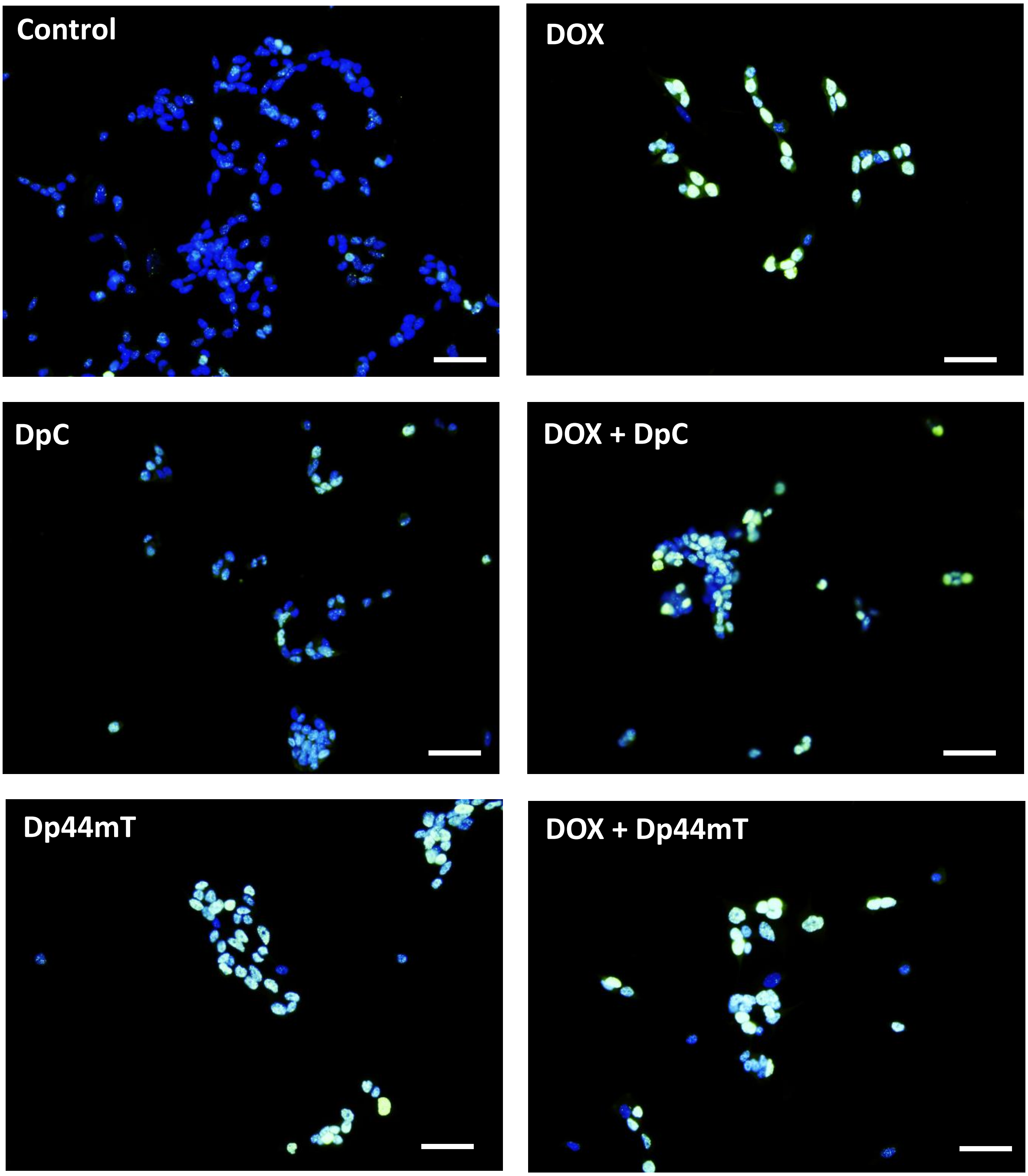

Figure S4: Detection of phosphorylated γ-H2AX as double-strand break marker in cell lines after the experimental treatment. Immunofluorescence was performer after 48 h treatment with IC_{50} doses of DpC, Dp44mT, DOX or their combinations. Phosphorylated γ-H2AX was visualized using the γ-H2AX primary antibody and Alexa 488-conjugated secondary antibody (green). The nuclei were labeled by DAPI (blue). Scale bars, 50 µm.

SUPPLEMENTARY FIGURE S4

(C) Saos-2 cell line

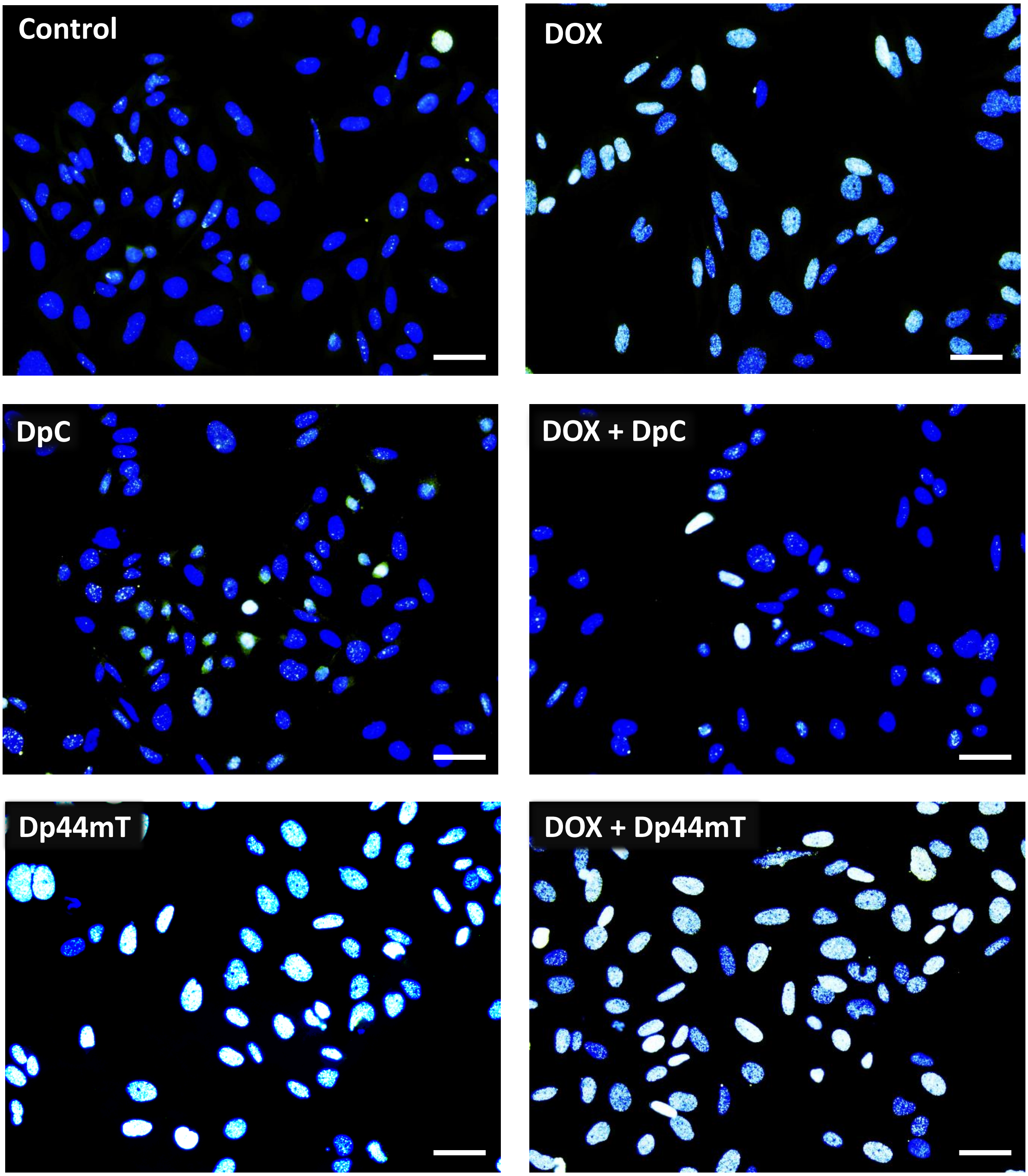

Figure S4: Detection of phosphorylated γ-H2AX as double-strand break marker in cell lines after the experimental treatment. Immunofluorescence was performer after 48 h treatment with IC_{50} doses of DpC, Dp44mT, DOX or their combinations. Phosphorylated γ-H2AX was visualized using the γ-H2AX primary antibody and Alexa 488-conjugated secondary antibody (green). The nuclei were labeled by DAPI (blue). Scale bars, 50 µm.

SUPPLEMENTARY FIGURE S4

(D) Daoy cell line

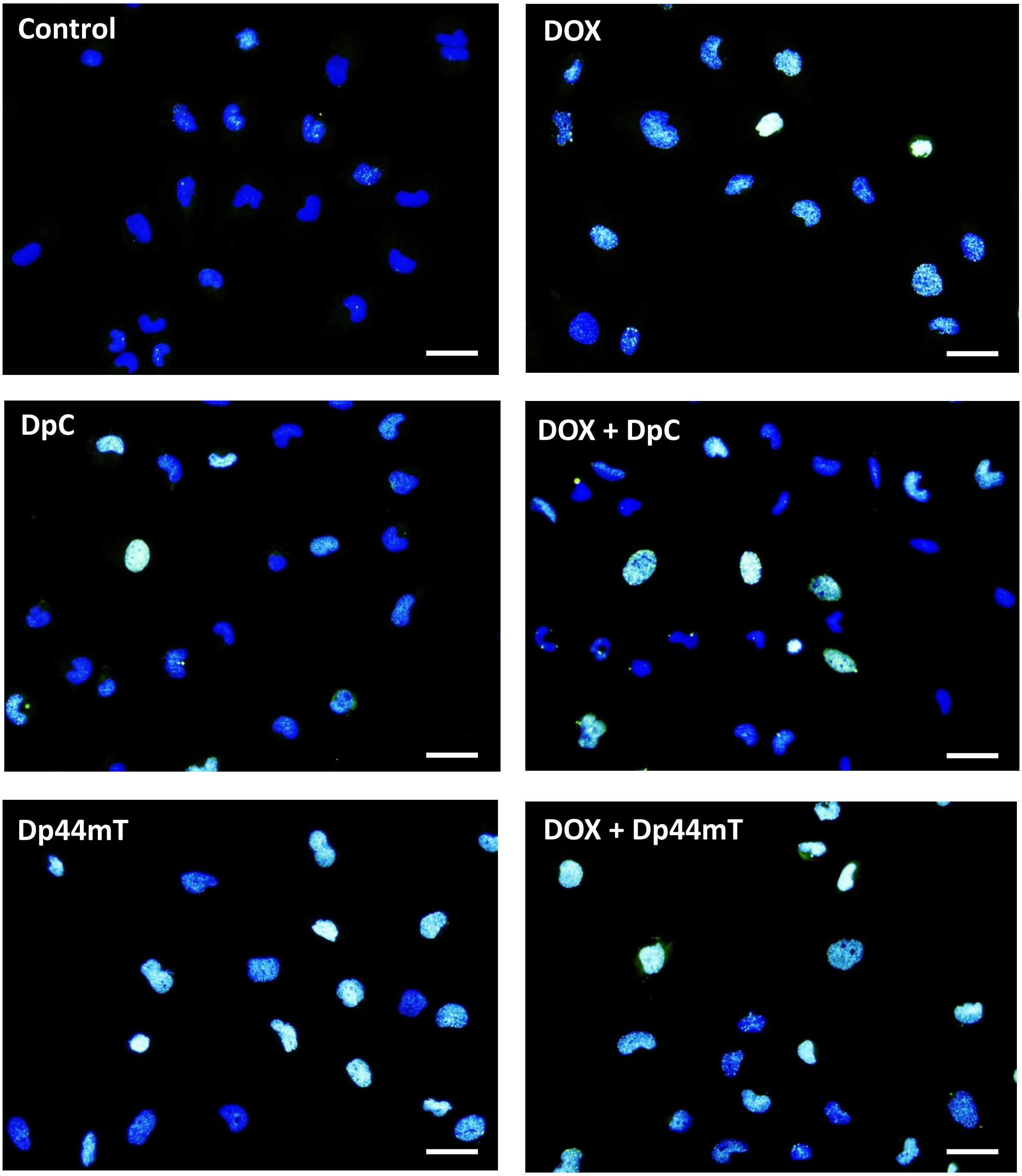

Figure S4: Detection of phosphorylated γ-H2AX as double-strand break marker in cell lines after the experimental treatment. Immunofluorescence was performer after 48 h treatment with IC_{50} doses of DpC, Dp44mT, DOX or their combinations. Phosphorylated γ-H2AX was visualized using the γ-H2AX primary antibody and Alexa 488-conjugated secondary antibody (green). The nuclei were labeled by DAPI (blue). Scale bars, 50 µm.

SUPPLEMENTARY FIGURE S4

(E) RD cell line

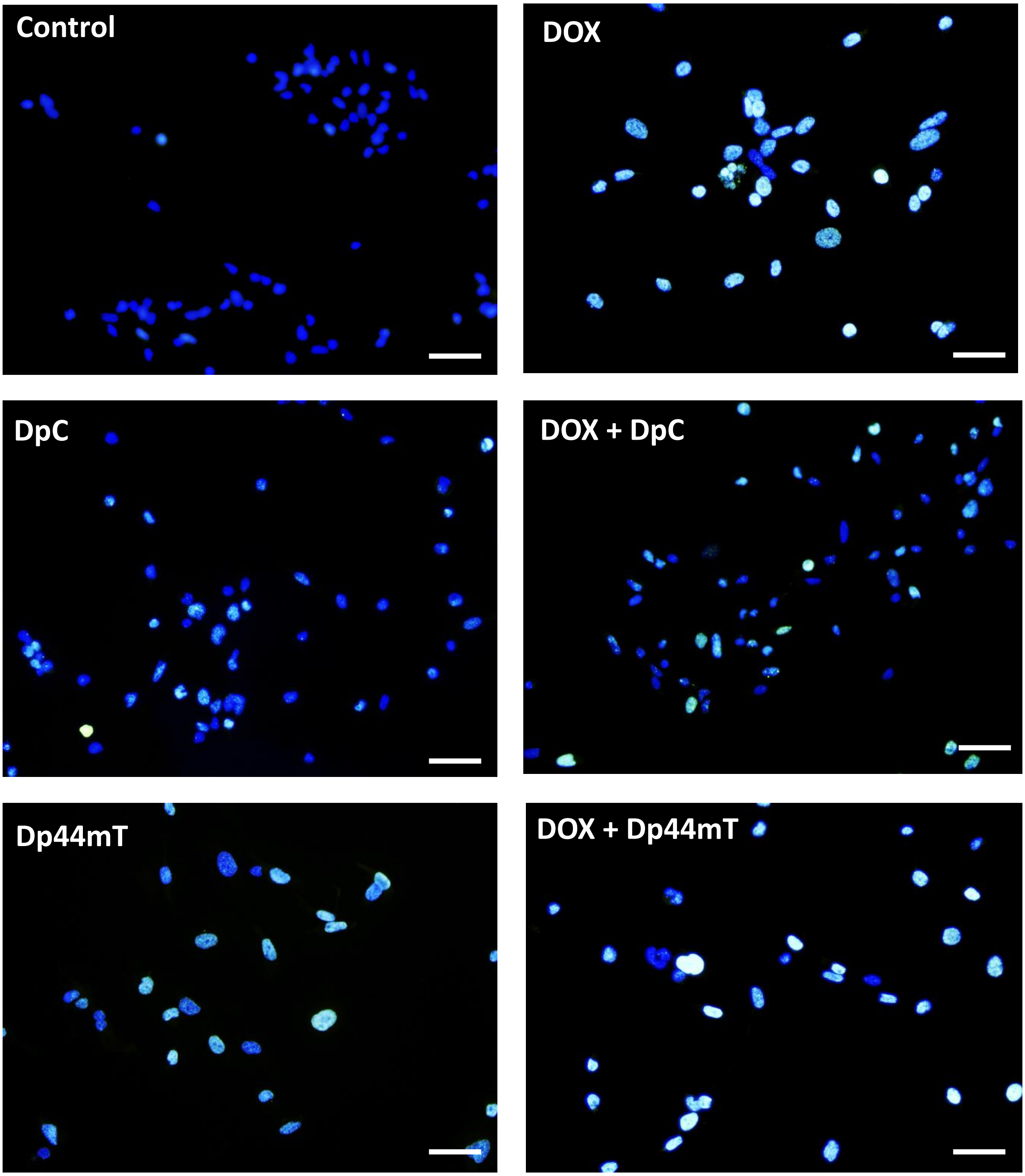

Figure S4: Detection of phosphorylated γ-H2AX as double-strand break marker in cell lines after the experimental treatment. Immunofluorescence was performer after 48 h treatment with IC_{50} doses of DpC, Dp44mT, DOX or their combinations. Phosphorylated γ-H2AX was visualized using the γ-H2AX primary antibody and Alexa 488-conjugated secondary antibody (green). The nuclei were labeled by DAPI (blue). Scale bars, 50 µm.

SUPPLEMENTARY TABLE S1

		Control [mean ± sd]	DOX [mean ± sd]	DAU [mean ± sd]	MIT [mean ± sd]
**SH-SY5Y**	**sub G_1**	3.2 ± 1.6	19.5 ± 3.4	32.6 ± 4.4	29.2 ± 7.3
	**G_0/G_1**	59.9 ± 6.0	42.7 ± 9.4	36.3 ± 9.7	41.9 ± 7.3
	**S**	11.0 ± 4.8	11.6 ± 3.1	12.3 ± 0.8	12.6 ± 2.4
	**G_2/M**	25.9 ± 4.9	26.1 ± 8.2	18.8 ± 5.5	16.3 ± 1.1
**SK-N-BE(2)**	**sub G_1**	2.7 ± 1.4	7.7 ± 2.4	13.6 ± 5.2	15.3 ± 0.9
	**G_0/G_1**	54.7 ± 0.5	13.7 ± 2.0	15.8 ± 1.8	24.3 ± 6.1
	**S**	13.8 ± 0.7	23.0 ± 13.2	19.4 ± 4.2	20.4 ± 1.6
	**G_2/M**	28.8 ± 1.0	55.6 ± 14.2	51.1 ± 3.2	40.1 ± 7.3
**Saos-2**	**sub G_1**	1.7 ± 0.7	18.7 ± 12.1	42.7 ± 3.1	23.1 ± 5.4
	**G_0 /G_1**	61.4 ± 2.7	16.5 ± 12.7	7.7 ± 0.3	9.3 ± 2.4
	**S**	14.0 ± 2.1	11.3 ± 1.5	10.7 ± 2.1	10.6 ± 1.0
	**G_2 /M**	22.9 ± 1.0	53.5 ± 12.6	38.8 ± 3.3	57.0 ± 5.1
**Daoy**	**sub G_1**	1.6 ± 0.5	25.5 ± 4.4	26.1 ± 7.3	26.0 ± 4.3
	**G_0 /G_1**	63.7 ± 2.2	14.7 ± 4.6	12.3 ± 3.3	16.0 ± 2.5
	**S**	16.2 ± 2.4	13.6 ± 0.6	18.0 ± 2.5	13.3 ± 1.9
	**G_2 /M**	18.5 ± 1.0	46.2 ± 8.4	43.6 ± 10.5	44.7 ± 2.3
**RD**	**sub G_1**	1.3 ± 0.1	13.4 ± 6.4	25.4 ± 15.7	18.7 ± 13.1
	**G_0/G_1**	55.5 ± 4.9	20.7 ± 17.5	23.7 ± 5.3	23.1 ± 3.6
	**S**	12.0 ± 2.8	11.6 ± 5.6	11.2 ± 2.2	11.0 ± 2.4
	**G_2/M**	31.2 ± 2.5	54.4 ± 25.4	39.7 ± 21.6	47.2 ± 18.9

Table S1: Cell cycle analysis in cell lines after the treatment with IC50 doses of anthracyclines. DNA content was measured after propidium iodide staining with subsequent flow cytometry detection. The changes in the cell cycle were obtained after 72 h of treatment with IC_{50} doses of doxorubicin (DOX), daunorubicin (DAU) or mitoxantrone (MIT). The table provides values representing mean ± sd (%) for each cell proportion in the cell cycle phases (sub G_1, G_0/G_1, S and G_2/M). All experiments were performed in biological triplicates.
